# A Comprehensive Overview on Stress Neurobiology: Basic Concepts and Clinical Implications

**DOI:** 10.3389/fnbeh.2018.00127

**Published:** 2018-07-03

**Authors:** Lívea Dornela Godoy, Matheus Teixeira Rossignoli, Polianna Delfino-Pereira, Norberto Garcia-Cairasco, Eduardo Henrique de Lima Umeoka

**Affiliations:** ^1^Physiology Department, Ribeirão Preto School of Medicine, University of São Paulo, São Paulo, Brazil; ^2^Department of Neuroscience and Behavioral Sciences, Ribeirão Preto Medical School, University of São Paulo, São Paulo, Brazil

**Keywords:** stress history, stress response, physical and psychological stressors, neuroanatomy, SAM axis, HPA axis, HPA axis time-domain, clinical implications of stress

## Abstract

Stress is recognized as an important issue in basic and clinical neuroscience research, based upon the founding historical studies by Walter Canon and Hans Selye in the past century, when the concept of stress emerged in a biological and adaptive perspective. A lot of research after that period has expanded the knowledge in the stress field. Since then, it was discovered that the response to stressful stimuli is elaborated and triggered by the, now known, *stress system*, which integrates a wide diversity of brain structures that, collectively, are able to detect events and interpret them as real or potential threats. However, different types of stressors engage different brain networks, requiring a fine-tuned functional neuroanatomical processing. This integration of information from the stressor itself may result in a rapid activation of the Sympathetic-Adreno-Medullar (SAM) axis and the Hypothalamus-Pituitary-Adrenal (HPA) axis, the two major components involved in the stress response. The complexity of the stress response is not restricted to neuroanatomy or to SAM and HPA axes mediators, but also diverge according to timing and duration of stressor exposure, as well as its short- and/or long-term consequences. The identification of neuronal circuits of stress, as well as their interaction with mediator molecules over time is critical, not only for understanding the physiological stress responses, but also to understand their implications on mental health.

## Introduction

Stress is recognized as an important issue in basic and clinical neuroscience research (de Kloet et al., 2005a; McEwen et al., 2015). Although this physiological phenomenon is fundamental to survival, it is also strongly related to several brain disorders including, depression, anxiety, post-traumatic stress disorder (Ruiz et al., 2007; Heim et al., 2008; Martin et al., 2009; Walsh, 2011; Saveanu and Nemeroff, 2012; Nemeroff, 2016) accordingly to the International Classification of Diseases, 10th edition (ICD-10). Research focusing on the complexity of stress is still challenging, but several studies in animals and humans have made substantial contributions to its progress in recent years, as reviewed by Hariri and Holmes (2015). However, it is crucial to provide more meaningful advances in the stress field by means of a translational approach, integrating basic knowledge and clinical practice.

In this review article, we briefly describe the history of stress research and highlight stress basic concepts, explore the complex neuroanatomy, in fact networks, of the stress system and its keen axes and mediators, as well as the time domains of the stress response. Lastly, we raise clinical implications from these concepts and discuss future research challenges on the stress field.

## History of Stress Research

In the beginning of the last century, the North American physiologist Walter Bradfort Cannon, after a series of investigations, made a compilation on the visceral adaptive responses to different stimuli. This compilation, which has been mainly conducted in physiology laboratories at Harvard University, described bodily changes occurring in conjunction with nociceptive events, hunger and cold, exercise and strong emotions. Cannon noted that functions that establish and support the body energy reserves at rest, in face of a stressful situation, were immediately intensified or completely interrupted in order to mobilize great energy. This mobilization allows an improvement of potential escape and attack and/or defense responses. From these observations, Cannon proposed what he called the “*fighting-or-flight*” response (Cannon, 1915).

A few years later, Cannon coined the term *homeostasis* based on the idea of *milieu intérieur* (internal medium) of Claude Bernard; Cannon wrote: *“the blood and other fluids surrounding cells constitute the internal environment with which occur direct exchanges of each cell, and this, must always be kept with parameters suitable for cell function, regardless of changes that may be occurring in the external environment.”* Cannon determined homeostasis as the ability of maintaining the physiological systems in direction of a dynamic equilibrium. The concept of homeostasis together with the *fight-or-flight* response has contributed for the foundation of stress research.

Hans Selye, yet as a medical student, found that patients suffering from different diseases often exhibited similar symptoms that could constitute a single syndrome. These evidences confronted him again when he was seeking for new hormones, from ovarian cow extracts at the Department of Biochemistry at McGill University in Montreal. In his study, the effects that were primarily attributed to an ovarian hormone were also observed after administration of various other extracts from several organs and toxic substances. The latter, regardless of the preparation method, induced the same changes: increased adrenal cortex size, gastrointestinal ulcers, as well as thymus and lymph nodes involution, therefore constituting the so-called “General Adaptation Syndrome” (Selye, 1936).

The General Adaptation Syndrome is characterized by a set of non-specific responses, which according to Selye, develops in three stages: (1) alarm phase, characterized by acute manifestations; (2) resistance phase, when the acute manifestations disappear; and (3) exhaustion phase, when first stage reaction may be present again or when it may occur the collapse of the organism (Selye, 1936).

In this context, it was designed as the basis of the stress theory and the concept of stress was built from the physical laws of Hooke. Selye was the first to define *stress* from a biological point of view as “*a nonspecific response of the body to any demand made upon it*” (Selye, 1950, 1976).

Scientists have discovered that the response to stressful stimuli is elaborated and triggered by the, now known, *stress system*, which integrates a wide diversity of brain structures that, collectively, are able to detect events and interpret them as either a real or a potential threat: *stressor* (Dedovic et al., 2009). Many research after this period has been done and promoted great knowledge to the stress field (McEwen et al., 2015).

**Figure 1:**
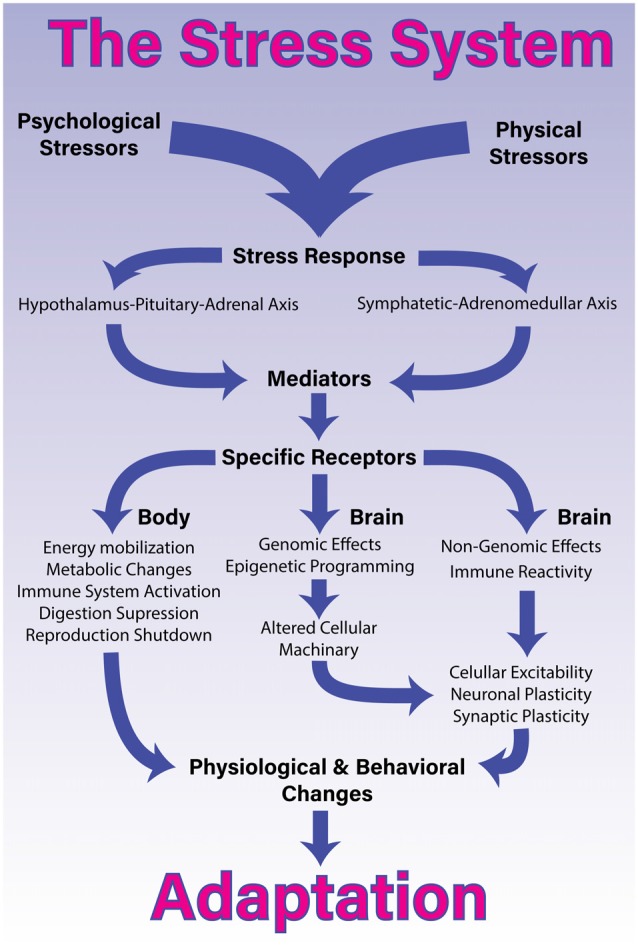
The stress system. Processing and coping with stressful situations requires the engagement of complex mechanisms that integrate brain and body. The response to stressful stimuli is articulated by a wide diversity of brain structures that collectively are able to detect or interpret events as either real or potential threats *(stressors)*. The perception of these events as stressors involves different networks depending whether it is a physical or psychological stressor. The identification of a stressor leads to activation of two major constituents of the stress system and the release of its final mediating molecules. The sympathetic-adreno-medullar (SAM) axis, secretes noradrenaline and norepinephrine and the hypothalamus-pituitary-adrenal (HPA) axis, secretes glucocorticoids. Once these axes are activated in response to a given stressor, they will generate a coordinated response that starts within seconds and might last for days, providing quick responses enabling both, an appropriated strategy, almost immediately, and homeostasis restoration. To accomplish this, the stress response systemically promotes energy mobilization, metabolic changes, activation of the immune system and suppression of the digestive and reproductive systems. More specifically in the brain, the stress response induces short- and long-term effects through non-genomic, genomic and epigenetic mechanisms. These central effects, combined with proinflammatory signaling, lead to alterations in cellular excitability as well as synaptic and neuronal plasticity. Collectively, these body-brain effects mediate alterations in physiology and behavior that enable adaptation and survival.

Thus, the perception of real or potential threats leads to the release of mediating molecules. The interaction between these molecules with their corresponding receptors, in the periphery and in the brain, results in the *stress response*, which through physiological and behavioral mechanisms restores the body homeostasis and promotes adaptation (de Kloet et al., 2005a; Joëls and Baram, 2009; Figure 1).

In the last few decades, the initial concepts of stress have been revised. Sterling and Eyer (1988) as well as others (McEwen and Stellar, 1993; Schulkin et al., 1994) proposed the new concept of *allostasis*, *allostatic load* and *allostatic overload*. These concepts would take into account the findings of the 60s, 70s and 80s from stress research, such as physiological variations in normal states and the presence of anticipatory responses to stressors (Dallman, 2003; Schulkin, 2003). The latter authors highlighted that the new concepts of allostasis, allostatic load and allostatic overload are more biologically precise than stress, to understand a complex system of adaptation or maladaptation. In the definition of allostasis, the set-point to maintain a physiological equilibrium is constantly changed, so what is ideal for baseline situations is not necessarily ideal in stressful situations (Koolhaas et al., 2010, 2011). Moreover, the set-point to maintain physiological balance in allostasis does not work in a linear fashion, but it can be regulated in several ways (McEwen and Karatsoreos, 2015). Finally, the terms “allostatic load” and “allostatic overload” consider the cumulative effects of stressors on the body in normal physiological and pathological situations, respectively (McEwen and Karatsoreos, 2015). Despite the proposal of the allostasis concept, some authors argue that the concept of homeostasis was interpreted in a restricted manner, and the use of the term allostasis would be just a semantic change, since the allostatic response system remains the same biologic system studied in homeostatic response (Dallman, 2003; Day, 2005). In fact, the proposed concepts of allostasis still offer little help to the understanding of stress, and rather than clarifying, brought several misunderstandings to the stress neurobiology (Davies, 2016). At last, the necessity of the allostasis concepts is questioned with indications that it is not necessary or desirable at this point on stress research (Dallman, 2003). In general, the concepts of stress, stressor and stress response are still widely used and accepted by the scientific community, and accordingly we adopt them in the current review.

## Neuroanatomy

The stress response involves an efficient, evolutionarily-conserved and complex system, with modulation in several levels of the central nervous system (CNS), governing learning, memory and strategic decision (Sapolsky and Pulsinelli, 1985; Sapolsky et al., 1985; Pavlides et al., 1993; Sapolsky, 2000a; McEwen, 2007; Joëls and Baram, 2009; Bains et al., 2015).

The first step in the stress response is the perception of a stressor. When a situation is perceived as a threat, the brain recruits several neuronal circuits to maintain physiological integrity even in the most adverse conditions (Ulrich-Lai and Herman, 2009). However, detection of different types of stressors requires engagement of different networks. Psychological and physical stressors engage different neuronal networks and cellular activity, leaving distinct footprints within the brain. Stimuli that produce actual disturbances of physiological status, that overwhelm the organism, e.g., hemorrhage or infection are considered as physical stressors. In the other hand, psychological stressors are generally defined as stimuli that threaten the current state and are perceived in an anticipatory condition e.g., aversive environmental stimuli, predator-related cues and failure to satisfy internal drives (Dayas et al., 2001).

**Figure 2:**
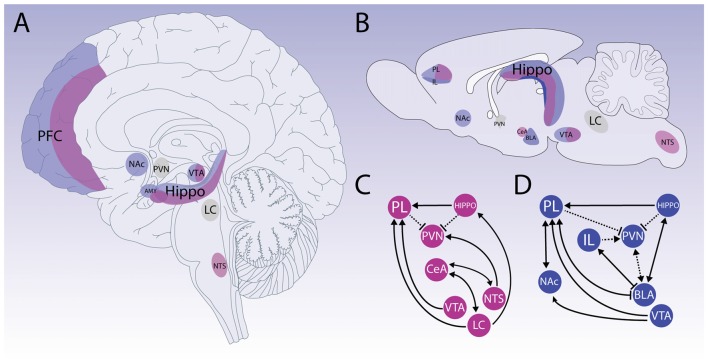
Neuroanatomy of stress. Schematic representation of primarily neuroanatomical substrates responsible for physical (pink) and psychological (blue) stressors processing. Upper panels show that neural processing for different types of stressors detection and appraisal of the situation engage several structures, which may overlap at some instances on human and rodent brain (**A,B**, respectively). Bottom panels represent how physical and psychogenic stressors require engagement of different networks (**C,D**, respectively). Physical stressors mainly activate structures related to vital functions control located on brainstem and hypothalamus. Structures such as the nucleus of the solitary tract (NTS) and *locus coeruleus* (LC) have an important role in the physical stress pathways. However, prosencephalic regions also participate in physical stress processing, such as prelimbic area (PL) in pre-frontal cortex (PFC). Also, it is important to mention that the central nucleus of the amygdala (CeA) participates in autonomic response integration. For instance, psychological stressors are perceived in an anticipatory condition, which may heavily rely on limbic structures and can be modulated by the reward system. The PFC is critical to develop appropriate responses to environment changes, and it is densely innervated by dopaminergic projections from the Ventral Tegmental Area (VTA) and Nucleus Accumbens (NAc). PFC disruption is associated with anhedonia and aberrant reward-seeking behavior. Although PFC involvement is complex and integrates different stress responses in general, PL and Infralimbic (IL) regions coordinate a top-down control. The amygdaloid complex also participates on psychological stress circuitry and with PFC disruption its involvement becomes more prevalent, and the circuitry switches to a bottom-up control. Another important structure that stands out due to its importance on cognitive and memory function, and that is activated in response to both physical and psychological stressors is the Hippocampus (HIPPO). The CA1 region has important connections with the above-mentioned limbic structures and HIPPO is an important structure of the HPA axis negative feedback. The paraventricular nucleus of hypothalamus (PVN) and LC (shown in gray) represent the main relay of the stress response triggering respectively the HPA and the SAM axis. The cross-talk activity between those nuclei allows a cognitive processing of the stress response and enables complex behavioral responses.

Therefore, physical and psychological stressors are processed by different circuitries in the brain, which may overlap at some instances. Regardless of the stressor processing, the stress system will be activated in a coordinated fashion. Here, we present up-to-date information on brain processing for physical and psychological stressors and how they interact (Figure 2).

### Physical Stressors

Physical stressors are mainly processed by brainstem and hypothalamic regions (Dayas et al., 2001; de Kloet et al., 2005a; Fenoglio et al., 2006; Joëls and Baram, 2009; Ulrich-Lai and Herman, 2009), they usually require immediate systemic reaction, which might be considered reflexive (Ulrich-Lai and Herman, 2009). Thus, the first phase of the stress response (sympathetic adrenomedullar system—SAM), provides a rapid physiological adaptation, resulting in short-lasting responses, such as alertness, vigilance and appraisal of the situation, enabling a strategic decision to face the challenge in the initial phase of a stressful event (de Kloet et al., 2005a; Joëls and Baram, 2009). Whereas, the secondary phase involves the hormonal mechanism (Hypothalamic Pituitary adrenal axis—HPA) considered sluggish compared to the synaptic mechanisms that activate the SAM, but resulting in an amplified and protracted secretory response (long-lasting responses). Both SAM and HPA axes will be further detailed in the below sections.

Thus, when a real stressor is recognized or perceived through signals (such as pain, inflammation and others) by the brainstem, the neurocircuitry that includes preganglionic autonomic neurons and hypophysiotrophic neurons in the paraventricular nucleus of the hypothalamus (PVN) are activated, generating a rapid autonomic nervous system (ANS) and the HPA axis responses (as illustrated in Figure 1; Ulrich-Lai and Herman, 2009).

In addition to several nuclei of the brainstem, medullary and spinal cord systems (that participate in the activation of ANS through its sympathetic and parasympathetic arms), physical stressors induce the activation of other brain structures that regulates autonomic stress response including PVN, nucleus of the solitary tract (NTS) and dorsomedial hypothalamus (DMH; Geerling et al., 2010). Such structures also have projections to both sympathetic and parasympathetic ANS arms.

The PVN contains distinct populations of neurons that project to autonomic targets in the brainstem and spinal cord (such as the middle cell column, the parabrachial nucleus, the DMX and NTS) and to median eminence sympathetic outflow therefore can be mediated by the PVN, but not exclusively. Complementary to the PVN role, another important hypothalamic nucleus is the DMH, which houses anatomically segregated neuronal populations responsible for regulating autonomic responses and also important for activating or inhibiting HPA axis activity (Ulrich-Lai and Herman, 2009).

The Locus coeruleus (LC; Cunningham and Sawchenko, 1988) and rostral ventrolateral medulla (RVLM; Dempsey et al., 2017) also directly innervate the intermediolateral cell column, while the dorsal motor nucleus of the vagus nerve (DMX) and the nucleus ambiguus (NA) mediate descending outputs for postganglionic parasympathetic nervous system (Saper and Stornetta, 2015).

It is well known that in the brainstem the NTS has an important role in the stress pathways. It is located in a quite strategic position and constitutes a relay for sensory, visceral and somatic information (Sapolsky et al., 2000; Herman et al., 2003; Ulrich-Lai and Herman, 2009; Bains et al., 2015). Moreover, the NTS is directly involved in the control of the cardiovascular and respiratory functions, containing different neuronal groups that receive distinct ascending pathways, as part of the baroreflex and chemoreceptors regulation (Machado, 2001; Zoccal et al., 2014) systems. The NTS also modulates the HPA axis activity mainly through noradrenergic and adrenergic projections to the PVN, but some studies indicate involvement of other catecholaminergic pathways in this modulation (Herman et al., 2003; Gaillet et al., 1991; Ritter et al., 2003).

Other important structures for triggering the stress response are the circumventricular organs (i.e., the median preoptic nucleus, the subfornical organ (SFO) and the organum vasculosum of the lamina terminalis), that respond to perturbations in fluid and send projections to other integrative brain regions. For instance, the activation of the medial parvocellular PVN by the SFO is essential for the central blood pressure regulation by angiotensin II and regulation of drinking behavior (Simpson and Routtenberg, 1975; Hindmarch and Ferguson, 2016).

And finally, it has also been described that limbic forebrain regions may contribute to physical stressors processing as well, influencing the functions in the autonomic responses to stress and the activation of the HPA-axis (Ulrich-Lai and Herman, 2009). These limbic stress circuits involve the amygdala, hippocampus and prefrontal cortex (PFC) that receive associational information from subcortical and cortical areas and their output converge to subcortical relay sites, making downstream processing of limbic information (top-down regulation; Ulrich-Lai and Herman, 2009). Those circuitries will be explored in the circuits overlapping section and we have highlighted how stressors might interact with the reward (approaching × aversion) system.

### Psychological Stressors

While stressors that are predominantly physically demanding are more likely to evoke autonomic stress responses, psychological uncontrollable and social-evaluative threatening stressors elicit both physical and cognitive stress responses (Skoluda et al., 2015). Together with the prosencephalic nuclei, components of limbic circuits such as the PFC, amygdala, hippocampus (HIPPO), PVN, ventral tegmental area (VTA) and nucleus accumbens (NAc) have a fundamental role in the regulation of stress response (Ulrich-Lai and Herman, 2009; Russo and Nestler, 2013).

The PFC is critical to develop appropriate responses to environment changes, enabling behavioral plasticity (Ridderinkhof et al., 2004). However, the involvement of PFC in stress response is complex, since different anatomic subdivisions play different roles. Bilateral lesions of prelimbic (PL) cortex portion increases plasma level of adenocorticotrophic hormone (ACTH), corticosterone and PVN c-Fos expression (Dioro et al., 1993; Figueiredo et al., 2003). However, lesions of infralimbic (IL) cortex reduce corticosterone secretion (Sullivan and Gratton, 1999). Moreover, studies with animal behaviors showed that lesions in dorsal sites of PFC result in anxiogenic effects, while lesions in ventral sites of PFC have anxiolytic effects (Sullivan and Gratton, 2002). These data indicate that PL and IL have opposite effects in psychological stressors response, inhibiting PVN activity with anxiolytic effects or stimulating PVN with anxiogenic behaviors, respectively (Radley et al., 2006; Jones et al., 2011).

Despite the functional connection between PFC and PVN, anatomical studies indicate that PFC only has indirect projections to PVN (Herman et al., 2003). The PL innervates several GABAergic neurons in the BST, which induces inhibition of PVN, whereas it is suggested that IL pathway projects to non-GABAergic in the BST to stimulate the PVN (Radley, 2012). However, it remains to be elucidated the exact pathways between PVN and PFC (Bains et al., 2015).

The PFC also has major projections to the amygdala, an important structure associated with emotional processing (Gabbott et al., 2005; LeDoux, 2007). Initially, the entire amygdala complex appears to promote synthesis and secretion of corticosteroids (Kawakami et al., 1968; Saito et al., 1989). However, subsequent studies showed that amygdala subnuclei have distinct roles in stress response (Roozendaal et al., 2009). The amygdala complex can be divided in the basolateral nucleus (BLA), the central nucleus of the amygdala (CeA) and the medial nucleus (MeA). Among these, the BLA has a major role in processing psychological stressors (Janak and Tye, 2015), being mainly activated by anticipatory stressors (Cullinan et al., 1995). However, BLA does not affect corticosterone release itself (Seggie, 1987). The role of BLA in the processing of psychological stressors seems to be more critical to the consolidation of aversive memories (Roozendaal et al., 2009).

Intra-BLA infusions of GABAergic receptor agonists impair aversive memory consolidation (Brioni et al., 1989). Besides, after emotional arousal, BLA neurons show lasting increases in the spontaneous firing rates, which could facilitate the consolidation of emotional memories through synaptic plasticity changes (Pelletier et al., 2005). Alterations in emotional and associative learning (i.e., aggression and learned helplessness) increased dendritic arborization in BLA neurons (Vyas et al., 2004; Bennur et al., 2007; Wood et al., 2008). In specific, some studies indicate that BLA-PFC pathway plays a major role in memory consolidation and stress response (Laviolette and Grace, 2006; Felix-Ortiz et al., 2016; Burgos-Robles et al., 2017). The BLA have extensive bi-directional monosynaptic glutamatergic projections with PFC (PL and IL; Gabbott et al., 2005; Dilgen et al., 2013; McGarry and Carter, 2016), which is crucial for emotional learning, fear-related responses, anxiety-like behaviors and social interactions (Laviolette and Grace, 2006; Felix-Ortiz et al., 2016; Burgos-Robles et al., 2017). Finally, the activity of stress response in the BLA is probably not only dependent on interactions with PFC, but also mediated by projections between PVN and amygdala (Prewitt and Herman, 1998).

The BLA has abundant output to the CeA and MeA, which have multisynaptic connections to the PVN (Herman et al., 2003). The CeA and MeA innervate brainstem structures that directly projects to PVN, such as the bed nucleus of the stria terminalis (BNST; Prewitt and Herman, 1998). However, recent evidence with optogenetics approaches indicates that PVN projections to the CeA are activated during retrieval of long-term fear memories. In contrast, retrieval of short-term memories is mostly dependent on PFC inputs to the BLA, indicating a time-dependent shift in fear memories circuits (Do-Monte et al., 2015). In turn, CeA has direct connections to periaqueductal gray (PAG), a fundamental area that organizes the threat response (LeDoux, 2012). Specifically, dorsal PAG is related to unconditioned threats exposure and ventral PAG is activated in conditioned threats, each one with distinct behavioral outputs. While lesions in dorsal PAG increased freezing behavior, damage to the ventral PAG decreased freezing (De Oca et al., 1998; LeDoux, 2012).

Another important limbic structure for stress processing is the hippocampal formation (McEwen et al., 1968; Smotherman et al., 1981), which consists of the dentate gyrus (DG) and Ammon’s horn/*Cornus Ammonics* (CA) subfields that compose the well-known tri-synaptic circuit, first from DG granule cells to CA3 pyramidal cells and then from CA3 to CA1 pyramidal cells (Amaral and Witter, 1989; Andersen et al., 2000; Naber et al., 2001). While the DG receives most of extra-hippocampal afferents from the entorhinal cortex via the perforant path (Witter, 2007), the CA1 and subiculum pyramidal neurons are the main HIPPO output to several areas, such as excitatory projections to PFC (PL and IL) and indirect inhibitory projections to PVN (Barbas and Blatt, 1995; Herman and Mueller, 2006).

The hippocampal inhibitory control over the HPA axis acts in a negative feedback manner inhibiting it (Jacobson and Sapolsky, 1991; Herman et al., 1996). The mainly indirect hypothalamic input of HIPPO originates in the CA1 and subiculum that densely innervate BNST and hypothalamic structures, except PVN (Canteras and Swanson, 1992; Cullinan et al., 1993).

Projections from HIPPO to both the PFC and BLA present some of the most important role in memory but especially for the regulation of psychological stress response (Godsil et al., 2013). HIPPO has bidirectional connections with BLA, but at the same time allows PFC to modulate the stress system with a top-down control (Mcdonald, 1998; Janak and Tye, 2015; Radley et al., 2015). However, the CA1 and subiculum monosynaptic glutamatergic efferents to PL and IL can become functionally disrupted after intense psychological stress (Zheng and Zhang, 2015). In a strong emotional experience, HIPPO and BLA rapidly activate memory-related mechanisms of synaptic plasticity while PFC functioning is suppressed, promoting long-lasting flashbulb memories (Diamond et al., 2007).

Therefore, in the presence of psychological stressors, if the activity of the amygdala and HIPPO becomes more prevalent, the stress system can switch to a bottom-up control (Arnsten, 2009). On top of that, prolonged psychological stress decreases glutamatergic projection to interneurons in BLA, leading to loss of BLA inhibition by the PFC and finally, to the hyperexcitability of BLA, which is responsible for behavioral abnormalities related to stress (Wei et al., 2017).

At this point, among several hypothalamic nuclei that are directly involved in regulating HPA axis and autonomic responses to stressors, the PVN stands out as the principal integrator of stress signals (Herman et al., 2003; Ulrich-Lai and Herman, 2009). In general, the medial parvocellular neurons of the PVN receive projections of first or second order of somatic afferent nociceptive, visceral, humoral or sensory pathways, beyond those of the limbic and reward system such as PFC, amygdala, HIPPO, VTA and NAc (Russo and Nestler, 2013; Bains et al., 2015). As already mentioned before, most limbic–PVN connections are indirect and are made through GABAergic cell groups in the BNST and peri-PVN regions of the hypothalamus (Crestani et al., 2013). Studies involving activity mapping (mostly c-Fos expression) have demonstrated an important role for BNST in the regulation of the HPA axis (Zhu et al., 2001), while the posterior BNST nuclei are involved in inhibition of the HPA axis, the anteroventral nuclei are involved in its excitation (Choi et al., 2007). In the reward system, pharmacological inhibition of NAc increased c-Fos expression in PVN (Noh et al., 2012). Lastly, the parvocellular neurons of the PVN are strategically connected with several brainstem and forebrain nuclei such as NTS, DMH, LC, VLM, DMX and NA, important to the processing and integration of both modalities of stressors, physical and psychological, which results in a rapid activation of the HPA axis (Ulrich-Lai and Herman, 2009; Senst and Bains, 2014).

### Overlapping Processing

Many of the above circuitry and brain areas responsible for physical and psychological stressors may sound exclusively related to brainstem and forebrain, respectively. However, some structures are also engaged with different modalities of stressor (Figure 2).

The best-characterized reward circuit in the brain is made up of dopaminergic neurons in the VTA that project to the NAc, a subdivision of the ventral striatum (Bath et al., 2017). The primary brain reward centers are inter-connected in complex ways in the brain reward circuitry, but mostly VTA and NAc regulates limbic areas mentioned above (Heshmati and Russo, 2015).

The dopaminergic tonus of VTA is fundamental to the recognition of rewards or punishment in the environment (Russo and Nestler, 2013). Exciting all VTA neurons enhances/mimics social defeat stress-induced effects on social avoidance and anhedonia while silencing all VTA neurons (regardless of the projection region or the cell type) promotes the opposite effect (Krishnan et al., 2007). On the other hand, the optogenetic stimulation of VTA dopaminergic neurons drives to a conditioned place preference (Tsai et al., 2009), whereas inhibition of VTA promotes conditioned placed aversion (Tan et al., 2012).

However, VTA is composed by different neuronal subpopulations, which participate in distinct circuits that encode different motivational signature (Lammel et al., 2012, 2014). Dopaminergic neurons of VTA project to NAc, HIPPO, BLA and PFC, however, the NAc also receives glutamatergic innervations from ventral HIPPO, BLA and PFC (Russo and Nestler, 2013; Lammel et al., 2014) and all these nuclei are under the regulatory influence of dopamine. Indeed, dopaminergic neurons that innervate the mPFC show reduced firing after social defeat chronic stress (Chaudhury et al., 2012). Dopaminergic neurons in VTA exhibit two patterns of spontaneous firing activity: a slow-frequency, single-spike firing and a burst firing, effectively regulating the activity of neurons in dopaminergic target areas and encoding reward-related signals (Grace et al., 2007). This mechanism of encoding and engaging specific networks makes sense since the behavioral response to a rewarding (approach) vs. an aversive experience (aversion) is different and therefore involves different, perhaps overlapping, neural circuits (Lammel et al., 2011).

Accordingly, associating this fined-tuned VTA regulation to the stress processing, it has been proposed that a specific ventral VTA pathway is related to painful physical stress. Several findings showed that dopaminergic cells that specifically project to the mPFC regulate the process of noxious stimuli (Abercrombie et al., 1989; Mantz et al., 1989; Bassareo et al., 2002; Brischoux et al., 2009; Matsumoto and Hikosaka, 2009; Lammel et al., 2011). But it is important to mention that although dopamine has a central role in processing physical stressors, in this scenario, the NAc detains the central role in regulating the reward system (Grace et al., 2007) which is deeply related to the processing of psychological stressors and promotion of behavioral flexibility (Heshmati and Russo, 2015).

Essentially, HIPPO projections to NAc promote susceptibility to psychological stress, BLA-NAc pathway increases reward seeking behavior and PFC-NAc promotes resilience (Stuber et al., 2011; Vialou et al., 2014; Bagot et al., 2015; Heshmati and Russo, 2015). Increased firing of VTA dopaminergic neurons that innervate the NAc occurs only in susceptible mice after chronic social defeat stress, while VTA-PFC pathway reduced firing after the same protocol (Chaudhury et al., 2012). Therefore, we can see that while mesocortical pathways encode physical processing, the NAc may represent an important hub in processing psychological stress.

Not only the PFC activity corresponds to an integrative hot point of limbic and reward circuitry, but also its contribution to the inhibition of the HPA axis by PFC top-down control is crucial. If the inhibitory HPA axis feedback does not function properly, as in the case of chronic stress and neuropsychiatric disorders, the loss of negative feedback in the PVN regulated by the PFC and HIPPO, associated to the hyperexcitability of BLA and silencing of NAc creates a positive feedback in the PVN that can both overload the stress system (Duvarci and Paré, 2007; Willner et al., 2013) and deplete the reward system (Heshmati and Russo, 2015).

Also, regarding the PFC, interesting data have revealed that stimulation of PL enhances hypoxia-mediated corticosterone responses and PVN by using c-Fos activation mapping (Jones et al., 2011). Some brainstem nuclei that regulate autonomic response, such as DMX and NAc, receive and send information from and to other regions of the CNS, respectively, including IL and PL portions of PFC, the CeA and BNST (Ulrich-Lai and Herman, 2009).

The CeA, previously mentioned as a central hub in fear response is also considered as a key node for stress integration (Ulrich-Lai and Herman, 2009). Moreover, NTS is also densely innervated by afferent projections from the CeA (Smith and Vale, 2006). The NTS can be activated during conditioning paradigms, suggesting that this pathway also plays a role in the integration of anticipatory stress (Pezzone et al., 1993).

It is important to highlight that different modalities of stressors that activate the PVN also activate the LC-Norepinephrine (LC-NE) system, such as restraint, shock (notably unpredictable), audiogenic stress, autonomic and immunological challenges and also social stress (Wood and Valentino, 2016). The LC is a compact pontine nucleus adjacent to the fourth ventricle and houses the majority of the NE-expressing neurons in the brain and innervates the entire neuraxis (Swanson and Hartman, 1975).

Activation of the LC-NE occurs in parallel and coordinated with PVN activation. This cross-talk between those nuclei allows a cognitive processing of the stress response engaging limbic and prosencephalic regions, such as the HIPPO and cortex, which govern neuronal excitability, cognition, memory and complex behaviors (Joëls and de Kloet, 1989; Wood and Valentino, 2016). Recent techniques, such as chemogenetics, optogenetics, combined with traditional retrograde tracing, which enable selective manipulation of LC-NE system in rodents, determined the pivotal role of the LC-NE, for example, for stress-induced anxiety-like behavior (McCall et al., 2015). Interestingly, Corticotrophin Release Hormone (CRH), which initially was discovered and defined as the hormone that initiates the cascade that ultimately leads to glucocorticoids release, seems to stand out as the molecule that coordinates the cross-talk between the two systems (Valentino and Van Bockstaele, 2008).

The LC-NE network is a major target of CRH (Chappell et al., 1986; Wood and Valentino, 2016). During stress, CRH is released into the LC, increasing neuronal firing rate and consequently NE release in forebrain targets (Valentino et al., 1991; Jedema and Grace, 2004). Different structures that coordinate autonomic and limbic processing such as CeA (Van Bockstaele et al., 1998), BNST (Van Bockstaele et al., 1999), PVN (Reyes et al., 2005), paragigantocellularis nucleus and Barrington’s nucleus (Valentino et al., 1992, 1996) send CRH modulatory projections to LC. Barrington’s nucleus is one of the few nuclei that actually sends projections directly into the core of the LC and also has important projections to preganglionic column of the lumbosacral spinal cord (Valentino et al., 1996).

Interestingly, CRH release within the LC is regulated by basal levels of corticosteroids as well (Valentino and Van Bockstaele, 2008).This communication between HPA and LC-NE axis determines the structural basis for emotional arousal, facilitates cognition and promote flexible behavioral responses to stress (Cole and Koob, 1988; Valentino and Van Bockstaele, 2008), since CRH release in the LC during stress facilitates shifting of attention between diverse stimuli (Snyder et al., 2012). Therefore, this crosstalk enables organisms to tailor different strategies to coping with constant changing environmental challenges.

## SAM Axis and Catecholamines

The stress response to physical and/or psychological stressors, organized by the previously described brain circuitry, involves a rapid physiological adaptation mediated mainly by catecholamines (de Kloet et al., 2005a; Joëls and Baram, 2009; Tank and Lee Wong, 2015). In fact, epinephrine (E) and NE are secreted from adrenal medulla (Cannon, 1914; de Kloet et al., 2005a; Joëls and Baram, 2009; Kvetnansky et al., 2009; Tank and Lee Wong, 2015) and NE from sympathetic nerves (Euler, 1946; Kvetnansky et al., 2009; Tank and Lee Wong, 2015).

The circuitry responsible for these autonomic modulations includes direct projections from PVN, LC and RVLM (Iversen et al., 2000; Ulrich-Lai and Herman, 2009) to pre-ganglionic sympathetic neurons present in dorsal intermediolateral cellular column (IML) of the spinal cord (thoracolumbar region), being these nuclei modulated by the NTS (Ulrich-Lai and Herman, 2009). Each pre-ganglionic fiber connects with many post-ganglionic neurons located in one or several pre-spinal ganglia or sympathetic paravertebral nuclei (Boron and Boulpaep, 2009). Although, there are many efferent pathways of the ANS, only two neurons are necessary to transmit impulses between the CNS to the effector tissue (Mccorry, 2007).

Other pre-ganglionic neurons, which end at the spinal cord, do not make synapses with a post-ganglionic neuron. For instance, they make synapses directly with chromaffin cells in the adrenal medulla (Mccorry, 2007; Boron and Boulpaep, 2009). This glandular tissue synthesizes and secretes E and NE, being E 80% of the catecholamine output by the adrenal medulla in humans (Goldstein et al., 2003; Dünser and Hasibeder, 2009; Tank and Lee Wong, 2015). Thus, these two components increase the capacity of the sympathetic division in influencing body visceral responses (Boron and Boulpaep, 2009). The sympathetic system activation leads to activation of signaling pathways that evoke changes in blood vessels, glands, visceral organs and smooth muscles (Tank and Lee Wong, 2015).

The parasympathetic component of the ANS regulates the action and duration of the autonomic responses, generating the so-called “vagal tone” of the cardiac and respiratory systems (Iversen et al., 2000; McEwen, 2007; Davidson and McEwen, 2012). Pre-ganglionic parasympathetic neurons originate from craniosacral vertebral segments (brainstem and sacral spinal cord) synapse with post-ganglionic neurons in the terminal nodes located more peripherally, and usually on the wall of the target organs. Post-parasympathetic ganglionic neurons stimulate the muscarinic and nicotinic receptors present on the membrane of the target cells by releasing acetylcholine (Boron and Boulpaep, 2009).

The effect caused by any of these substances, acetylcholine, norepinephrine and epinephrine, depends on the biochemical properties of the cells and on the receptor distribution in a determined tissue (Mccorry, 2007). In general, SAM activation is considered to mediate short-term effects, with rapid responses, while the HPA axis activation leads to short and long-term effects (Joëls and Baram, 2009; Tank and Lee Wong, 2015). As previously mentioned, interactions of these major stress systems (SAM and HPA) occur at several levels, functioning cooperatively and/or sequentially, acting in opposite ways in most visceral organ targets.

Although, these sympathetic and parasympathetic systems act independently from each other (Antunes-Rodrigues et al., 2005; Ulrich-Lai and Herman, 2009; Tank and Lee Wong, 2015) dominance of the sympathetic system occur under conditions such as exercise and “fight-or-flight” reactions, the parasympathetic system predominates during resting conditions (Mccorry, 2007).

E and NE interact with adrenergic receptors present in cell membranes of smooth muscles and in numerous organs, as well as in neurons widespread in the CNS (Mccorry, 2007; Tank and Lee Wong, 2015). These receptors are the G-protein coupled receptors (GPCRs), which are homologous to muscarinic receptors, i.e., are proteins embedded in the cell membrane with seven transmembrane domains (Mccorry, 2007; Dünser and Hasibeder, 2009). There are two major types of adrenergic receptors; α- adrenergic and β-adrenergic receptors, with their subtypes (Langer, 1980; Guimarães and Moura, 2001; Dünser and Hasibeder, 2009).

The rise in circulating E and NE causes general physiological changes that prepare the body for “fight-or-flight” reaction (Cannon, 1915, 1929; Bowman, 1930). Their effects include: maintaining alertness, metabolic actions (increased glucose via glycogenolysis and gluconeogenesis, lipolysis, increased oxygen consumption and thermogenesis) and cardiovascular actions (Aires, 2012).

The central noradrenergic system, specifically the LC is involved in multiple neurochemical circuits, having connections with neuroanatomical structures involved in the stress response, such as the HIPPO, amygdala and temporal neocortex. Studies indicate an important role of the LC in response to acute stress (Myers et al., 2017), acting as an “alarm system,” in attention, excitation and defensive responses (Cassens et al., 1981; Valentino and Van Bockstaele, 2008). However, chronic activation of the LC may have a potential role in the development of pathological behaviors related to stress (Southwick et al., 1999; Ziegler et al., 1999; Valentino et al., 2012; George et al., 2013; Reyes et al., 2015), which will be discussed in one of the following sections.

Thus, the release of NE has central actions, coordinates and modulates autonomic, endocrine and neuroendocrine responses, through extensive brain and spinal cord connections, while direct projections from LC to medial parvocellular division of the PVN (Cunningham and Sawchenko, 1988) allows the modulation of the HPA axis (Armario et al., 2012). On the other hand, the activity of LC itself may be influenced by CRH through afferent projections from amygdala and brainstem nuclei (McCall et al., 2015; Sun et al., 2015), facilitating the complex behavioral and cognitive reactions to stress (Valentino and Van Bockstaele, 2008).

## HPA Axis and Glucocorticoids

When an organism faces a threatening stimulus, whether it is psychological or physical, as explained in the previous sections, many brain areas are activated in a coordinated fashion to recruit a complex structure known as hypothalamus, which is composed by many sub nuclei. As already discussed, among those nuclei is the PVN, which is responsible for eliciting the activation of the HPA axis, one of the main components of the stress response. The PVN synthesizes three different neurochemical compounds that behave either as neurotransmitters or hormones, depending on where they are acting. These compounds are oxytocin, vasopressin and CRH (Vale et al., 1981; Sawchenko et al., 1992, 1996).

The pituitary is situated caudally and above the optical chiasm, and consists of an anterior portion (adenohypophysis), that synthesizes and secretes hormones and of a posterior portion (neuro-hypophysis), that stores oxytocin and vasopressin synthesized by the parvocellular neurons in the PVN (McCann and Brobeck, 1954; Joëls and Baram, 2009).

When CRH reaches the anterior pituitary, it stimulates the corticotrophs to synthesize and release the ACTH (Vale et al., 1981). ACTH is secreted through the hypophyseal portal system, and acts on the cortex of the adrenal gland, more specifically on the middle layer named *fascicullata* (Vale et al., 1978; Herman et al., 2003), which is responsible for glucocorticoid synthesis and secretion (Vale et al., 1978). The main glucocorticoid in humans is cortisol, and its equivalent in rodents is corticosterone (de Kloet, 2013).

Although the HPA axis has an ultradian rhythm (Young et al., 2004; de Kloet and Sarabdjitsingh, 2008; Lightman and Conway-Campbell, 2010), levels of corticosteroid hormones follow a circadian rhythm, as well as a glucocorticoid peak secretion in humans, occurring during the early morning and in rodents in the beginning of the evening (Reppert and Weaver, 2002). There are many evidences indicating that the suprachiasmatic nucleus (SCN) of the hypothalamus is the generator of the circadian rhythm (Welsh et al., 1995; Jagota et al., 2000; Reppert and Weaver, 2002; Engeland and Arnhold, 2005). Glucocorticoids act on the anterior pituitary, PVN and other brain structures, such as the HIPPO (McEwen et al., 1968), controlling the activity of the HPA axis. This phenomenon is called short and long negative feedback loops, respectively (de Kloet et al., 1991; Joëls and Baram, 2009; Bains et al., 2015).

Glucocorticoids are steroids and easily trespass cell membranes and since they are released in the bloodstream they can virtually reach any cell in the body. Although peripherally the glucocorticoids act massively in several target-organs, here we focus on their effects on the brain. Cortisol, or corticosterone in rodents, exerts their effects in the brain by binding to two types of receptors, the glucocorticoid receptor (GR) and the mineralocorticoid receptor (MR; Reul and de Kloet, 1985; de Kloet et al., 2005a,b). These receptors mediate the effects of glucocorticoids in the brain through genomic and non-genomic mechanisms (Verkuyl et al., 2005; de Kloet and Sarabdjitsingh, 2008; Groeneweg et al., 2012; Joëls et al., 2012) and therefore GR and MR characteristics, such as distribution, affinity and mechanism of action are determinant to regulate homeostasis under basal condition or to promote adaptation through the stress response.

The MR shows 10 times higher affinity for corticosterone than GR (Reul and de Kloet, 1985). Such difference is translated to receptor occupancy throughout the day, during the through phase of the circadian cycle, when glucocorticoid levels are low, MRs are occupied whereas the GRs are mostly free. During the peak phase of the circadian cycle, or after high glucocorticoid release due to the stress response, MRs are completely occupied while GRs are partially occupied (Kitchener et al., 2004; Young et al., 2004).

Although glucocorticoids are able to reach all neurons in the brain, they exert effects on those neurons expressing GRs and MRs. GRs are abundant and widely spread throughout the brain. On the other hand, MRs are expressed in restricted areas of the brain (Reul and de Kloet, 1986). It is also important to mention that there are some key structures that express both receptors, such as the PVN, HIPPO, amygdala, lateral septum, LC and NTS, among others. Moreover, GR and MR also co-localize with adrenoreceptors allowing the interplay between the SAM and HPA axes (Härfstrand et al., 1986; Joëls and de Kloet, 1989; Pu et al., 2007; Krugers et al., 2012; Zhou et al., 2012).

Upon binding to GR or MR, located in the cytosol, receptor-ligand complex forms monomers or dimers (homo or heterodimers) that translocate to the nucleus and bind to promoter regions of about 1%–2% of the genome (known as Glucocorticoid Responsive Elements), or interact with transcriptional factors, usually hampering their efficacy (Morsink et al., 2006; Datson et al., 2008; Grbesa and Hakim, 2017; Weikum et al., 2017). These delayed and long-lasting effects of GR and MR receptor activation (Joëls et al., 2012) mediate transcription of proteins involved in immune, cognitive, metabolic and many other physiological processes, in order to promote changes in physiology and behavior. Moreover, it has also been shown that not only genomic processes are triggered by GR and MR, actually there are studies clearly showing rapid effects of membrane-located MR and GR activation (Borski, 2000; Johnson et al., 2005; Karst et al., 2005; Olijslagers et al., 2008; Wang and Wang, 2009; Evanson et al., 2010; Karst et al., 2010; Roozendaal et al., 2010; Groeneweg et al., 2012; Nahar et al., 2016).

Important to mention, it has been demonstrated that GR levels, and consequently HPA axis function, can be modulated by the environment, as well as life experiences (acute and chronic stressors), through stable changes in the DNA chromatin, which does not alter DNA sequence, a mechanism known as epigenetics (Hunter et al., 2009; Griffiths and Hunter, 2014; Buschdorf and Meaney, 2015). Alterations such as methylation and acetylation of histones occur genomewide enhancing or hampering chromatin activity, however DNA methylation can also occur in a gene-specific fashion altering its expression (Tsankova et al., 2006; Nestler, 2014). In fact, Weaver et al. (2004) showed that environment factors, such as maternal care, play a fundamental role in the programming of the HPA axis function, morespecifically on the nuclear receptor subfamily 3 group C member 1 (*NR3C1)* gene (GR), which persists through adulthood. These authors demonstrated that pups, which received low care (licking and grooming the pups) from their dams, had higher hippocampal *NC3R1* methylation associated with lower GR expression, when compared to those that received high levels of licking and grooming.

Epigenetic modulation of GR expression has also been described in humans as highlighted by Palma-Gudiel et al. (2015) and Smart et al. (2015) and although some studies point out the fact that stress (early-life adversities) lead to *NR3C1* hypermethylation, it is still controversial the association of these epigenetic changes with psychopathologies such as depression in humans (McGowan et al., 2009; Alt et al., 2010; Na et al., 2014; Palma-Gudiel et al., 2015; Smart et al., 2015). In the other hand, pre-clinical data have associated stress-induced epigenetic alterations with vulnerability or resilience to psychiatry-like conditions (Covington et al., 2009, 2011; Wilkinson et al., 2009; Nestler, 2014).

## Stress and the Immune System

As mentioned previously, it is through the GR receptor that the HPA axis modulates the immune system, which involves protein-protein and/or DNA interactions (for further review see Liu et al., 2014). Although, the discovery of the interplay between the inflammatory and endocrine systems is dated as a long-time story, it still remains as a hot topic in the field of stress research. At the same period Hans Selye discovered his canonical findings on stress, the prestigious Mayo Clinique developed the substance E (Neeck, 2002; Hillier, 2007; Lupien, 2015). This substance crystalized by Dr. Kendall (Mason et al., 1936) was being used for treating Addison’s disease (Kendall, 1953), later on recognized as cortisol, which gave birth to a myriad of “wonder” drugs for a variety of inflammatory disorders such as Rheumatoid Arthritis (Neeck, 2002; Hillier, 2007; Lupien, 2015).

Most recently, research on the relationship between stress hormones and immune system has unraveled many intricate pathways that also can be explored for their clinical implications. It has been postulated that besides preparing the body to deal with the environment demands, stress activates the immune system, which engages active defense against physical injury and pathogens. Ultimately, cytokines are produced to promote multiple kinds of inflammatory responses (Takahashi et al., 2018). But reports stated that stress-enhanced inflammatory activity is present in the absence of infectious pathogens, especially in depressed patients (Audet et al., 2014).

To address whether stress or its hormones are pro or anti-inflammatory, evidence using rodent models demonstrated that stress itself can be both pro- and anti-inflammatory. Some authors have proposed that the timing of immune challenges and measurements determines the direction of glucocorticoid actions. Those authors proposed that glucocorticoids initially present anti-inflammatory action, but later on sensitizes the immune response on the recovery phase (after stressor; Frank et al., 2013).

Stress can directly influence immune signaling in two main ways, by reducing the inhibitory effects of glucocorticoid actions, or by directly stimulating the immune system via HPA axis and SAM (Liu et al., 2014; Wohleb et al., 2015). But not only the neural components of stress engage the immune system, the immune system also affects the CNS, modulating the HPA axis (Berkenbosch et al., 1987; Linthorst et al., 1994; Angeli et al., 1999). Therefore, acutely, stressful experiences enhanced levels of circulatory pro-inflammatory cytokines (Steptoe et al., 2007). Some of these inflammatory cytokines are either locally produced by activated microglia (Wohleb et al., 2015) or get access to the brain through circumventricular sites (Vitkovic et al., 2000) or are transported (Banks, 2006).

Interestingly, the sites that have increased pro-inflammatory immune reactivity appear to be related to acute stressor modalities. Social stressors increased expression of pro-inflammatory interleukins and activated microglia in sites such as PFC, amygdala and HIPPO (Audet et al., 2010, 2011; Tynan et al., 2010; Wohleb et al., 2011; Hinwood et al., 2012, 2013) whereas physical stressors promoted an increase in the hypothalamus (O’Connor et al., 2003; Deak et al., 2005; Hueston and Deak, 2014).

## Time Domain of Stress

The complexity of the stress response is not restricted to neuroanatomy and mediator molecules, but also diverge according to timing and duration of stressor exposure, as well as its short- and/or long-term consequences (de Kloet, 2013). Stress mediators operate in a feedback loop after HPA axis activation, and regulate, in a positive or negative way, different brain structures to restore homeostasis (de Kloet et al., 2005b). When the timing of stress response is inappropriate, aberrant HPA axis activity could lead to pathological states (Heim et al., 2000, 2008; de Kloet et al., 2005a; Juruena, 2013; Nemeroff, 2016). We can divide basal regulation of glucocorticoids release in ultradian and circadian cycles, and also categorize the effects of stress response in rapid or delayed regarding their initiation, and in short or long-term, regarding their duration, in both scenarios they range from milliseconds to days (Joëls et al., 2012). Moreover, stress also differs when it occurs in early-life or adulthood of an individual, which can increase or decrease the possibility of developing brain disorders (Lupien et al., 2009; Juruena, 2013; Timmermans et al., 2013).

In ultradian and circadian cycles, peaks of corticosterone release contribute to regulation of basal metabolic demand and the responsiveness of stress (Lightman and Conway-Campbell, 2010). The origin of pulsatile corticosterone release in an ultradian and circadian fashion is not fully elucidated, but it has been a general assumption that the hypothalamus modulates this phenomenon, specifically, the hypothalamic SCN has efferent projections to neuroendocrine cells in the PVN that trigger the HPA axis activation (Engeland and Arnhold, 2005).

Acute stress mediators start acting within seconds after the stressor detection and provide quick responses to an appropriated strategy, involving modulation of limbic-cortical circuits (Bains et al., 2015). The PVN in acute stress is fundamental, both to drive “fight-or-flight” responses through stress mediators release, or to inhibit acute response (Dallman, 2005). After corticosterone is released, frequency of miniature excitatory postsynaptic currents (mEPSCs) in PVN are suppressed, which decreases glutamatergic excitability and increases GABA inhibition (Di et al., 2005). These effects of corticosterone in the PVN occur mainly via non-genomic GR activity and endocannabinoid signaling (Di et al., 2003; Verkuyl et al., 2005). However, in other structures, excitatory activity is increased after acute stress (Joëls et al., 2012), for instance corticosterone release enhances mEPSC frequency via MR in CA1 pyramidal cells (Karst et al., 2005; Olijslagers et al., 2008). Similarly, mEPSC frequency in BLA is also increased via MR after acute corticosterone release (Karst et al., 2010). Interestingly, firing frequency of BLA neurons remains at high levels even after corticosterone washout, mainly modulated by GR and cannabinoid receptor 1 (Karst et al., 2010). In cortical structures, such as the PFC, acute stress situation increases GR-dependent glutamate release (Musazzi et al., 2010).

Hours after stressor exposure, delayed effects start to occur in a different way on limbic-cortical structures (Joëls et al., 2012). Neurons of CA1 have enhanced amplitude but not frequency of mEPSCs via GR (Karst et al., 2005; Martin et al., 2009). The GR activity also modulates synaptic plasticity in CA1, promoting Long-term Depression (LTD) and impairs Long-term Potentiation (LTP; Pavlides et al., 1993; Xiong et al., 2004; Kim et al., 2006). In opposite, corticosterone presents delayed effects via MR by increasing the induction of LTP in CA1 (Pavlides et al., 1996). In BLA, delayed effects of corticosterone increase excitability of neurons, maintaining excitability after acute stress (Duvarci and Paré, 2007). Similarly, corticosterone enhances glutamatergic transmission and reduces inhibitory post-synaptic currents (mIPSCs) in the PFC (Hill et al., 2011). These delayed effects of stress in limbic-cortical structures restore homeostasis, as well as retain important information to better cope with similar situations in the future (Joëls et al., 2013).

When there is an over-exposure to stressors, lasting from hours to days, it is possible to observe structural changes in limbic-cortical areas and even in the reward system (Joëls et al., 2013; Russo and Nestler, 2013). Dendritic complexity is progressively reduced in HIPPO and PFC, after chronic exposure to stressors (McEwen and Magarinos, 1997; Holmes and Wellman, 2009). By contrast, neurons in the BLA and NAc increase dendritic density, increase excitatory tone and decrease inhibitory tone in this context (Vyas et al., 2002; Christoffel et al., 2011; Muhammad et al., 2012). At the cellular level, chronic stress impairs induction of LTP in CA1 of the HIPPO and reduces both AMPA and NMDA-mediated synaptic transmission (Joëls et al., 2012; Yuen et al., 2012). The behavioral consequences of these structural changes were associated with anxious behavior, probably by hypertrophy of the amygdala (Mitra and Sapolsky, 2008) and deficits in learning, which could be explained by impaired hippocampal and PFC structures (Joëls et al., 2012; de Kloet, 2013) and reduced BDNF in VTA (Krishnan et al., 2007).

It is interesting to note that when chronic stress is experienced early in life, its effects on the brain last longer than when it occurs during adulthood (Lupien et al., 2009). When rodent pups are exposed to prolonged maternal separation, the density of CRH binding sites increases in HIPPO, amygdala and PFC (Anisman et al., 1998). Activity of CRH mediates stress-related synaptic plasticity loss in the HIPPO, anxiogenic behavior dependent of amygdala and cognitive impairment associated with PFC (Schulkin et al., 1998; Sánchez et al., 2001; Fenoglio et al., 2006).

## Clinical Implications

At this point, in our evolutionary history, stress could be implied in a maladaptive performance in a large proportion of the population, considering the large number of comorbidities that occur from dysfunction of the stress system (de Kloet et al., 2005b). The HPA axis dysregulation and prolonged exposure to glucocorticoids reduce the ability of neurons to resist insults, increasing the risk for injury by other toxic events (Lupien et al., 2009). Moreover, new researches have called attention to adversities in early life, which are greatly associated with higher vulnerability to disorders later in life, causing a long-term impact in the circuitry responsible for cognitive and emotional function (Gold et al., 1988; Heim and Nemeroff, 2002; Lippmann et al., 2007; Chrousos, 2009; Lupien et al., 2009; Juruena, 2013; Lucassen et al., 2013; Krugers et al., 2017).

In this sense, both basic and clinical researches have advanced in recent years but much remains to be understood about the subject. In general, animal models have provided a comprehensive view of the stress effects on the brain, abundantly on the limbic structures (Hariri and Holmes, 2015). The amygdala is a highly conserved brain structure that is fundamental to detect potential danger (Janak and Tye, 2015), while HIPPO provides support to encoding environmental information associated with the stressor (Herman et al., 2005) and the PFC provides associations between cues and stressor (Milad and Quirk, 2012). The identification of these highly evolutionary conserved networks that are affected by stress, allowed important discoveries in clinical research (Hariri and Holmes, 2015).

Increasing data highlight that highly debilitating stress comorbidities such as depression, anxiety disorders, PTSD and epilepsy share pathogenic mechanisms with stress dysfunction and between each other. These mechanisms are probably deeply connected and the structural and functional change caused by one disease triggers the other, despite these it is still not clear on the relationship between them (Gold et al., 1988; de Kloet et al., 2005a,b; Kanner, 2012). Considering this, we presented in this review a short glimpse of how stress is related to these CNS pathologies. For further review, we suggest to look into the literature cited here.

A significant percentage of patients with Major Depression (MD) have increased concentrations of cortisol in plasma, urine and cerebrospinal fluid, exaggerated cortisol response after ACTH hormone stimulation and hyperplasia of the pituitary and adrenal glands (Gold et al., 1988; Juruena et al., 2004). In addition, one of the most consistent findings in neuropsychiatry is the reduction of hippocampal volume by 10%–15% in MD (Sapolsky, 2000b).

Chronic stressors in early life result in permanent epigenetic, endocrine, neural, immune and inflammatory changes, constituting a relevant risk factor for several neuropsychiatric diseases in adult life (Xiong and Zhang, 2013; Zhang et al., 2013; Berens et al., 2017). Traumatic childhood experiences such as abuse, neglect and parental loss increase the incidence of psychiatric disorders, such as MD that boost to 59%–75% in adult life (Widom et al., 2007) and anxiety disorders that are 1.9–3.6-fold more common in people who experience early life stress (Fernandes and Osório, 2015). In epilepsy, stress can influence in multiple ways, often as seizure-precipitating but also increased the risk of epilepsy development (van Campen et al., 2014). Additionally, the dysregulation of HPA axis in stress can trigger immunological responses that are considered as risk factor for Alzheimer’s disease (Jeong et al., 2006; Caruso et al., 2018; Hoijemakers et al., 2016).

However, studies indicate that infancy coincides with a period referred as hyporesponsive period to stress (Stress Hyporesponsive Period—SHRP), which is supposed to be a period that is necessary for the proper development of the brain after birth (Sapolsky and Meaney, 1986; de Kloet et al., 2005b). It corresponds with a period of low peripheral concentration of glucocorticoids, in which a physiological response to mild stress (increased glucocorticoids and adrenaline, increased cardiovascular circulation, immune system modifications) does not occur. There is no consensus when exactly this period starts or how long it lasts, but it is suggested that it could last from around the 2nd to 12th postnatal day in rodents (Sapolsky and Meaney, 1986) and up to 5 years in humans (Gunnar and Donzella, 2002; Curley et al., 2011).

Interestingly, psychological (Sapolsky and Meaney, 1986) and multimodal stressors (Godoy et al., 2018) can disrupt the SHRP, turning the HPA-axis responsive to stressors earlier in development (Cowan et al., 2016) and generating disruptions related psychopathologies (Loi et al., 2014; Bath et al., 2017).

The effects of stress during early life (ELS) on the brain have been deeply studied (for review see Lupien et al., 2009; Lucassen et al., 2013; Krugers et al., 2017). ELS disrupts the proper development and function of limbic structures, leading to lifelong susceptibility to stress on behavior and cognition as well as on the reward system (Peña et al., 2017). More recently it has been demonstrated that ELS led to an early emergence of timed developmental suppression of fear behavior that correlates to an early maturation (Bath et al., 2016).

Depression-anxiety comorbidity is strongly associated with impairment in health, as well as in cognitive and emotional functions (Kroenke et al., 2007). Similarly, chronic treatment with corticosterone not only generates depressive-like symptoms but also induces amygdala hypertrophy and increases anxiogenic behavioral responses (Mitra and Sapolsky, 2008). In humans, individual differences in amygdala reactivity to threat-related facial expressions predict vulnerability to stress, such as subjects with hyperactivation of the amygdala are more likely to experience depression and anxiety symptoms (Yang et al., 2008; Swartz et al., 2015).

Also, hyperactivation of LC-NE is related to neuropsychiatric disorders such as PTSD and MD, the activation of this system out of proper context may lead to hyperarousal, loss of concentration, restlessness and impaired focused attention, which are characteristic symptoms of stress-related psychiatric disorders (Southwick et al., 1999; Wong et al., 2000). For instance, PTSD is associated with increased rates of anxiety and depression (Nemeroff et al., 2006). However, some clinical investigations reported low levels of cortisol in PTSD patients, while individuals with anxiety disorders or depression show an increase of cortisol response (Daskalakis et al., 2013; Zoladz and Diamond, 2016; Zorn et al., 2017).

Actually, the literature reports alterations in the HPA axis in PTSD patients and from the study by Mason et al. (1986), in which low basal levels of cortisol were detected in PTSD patients, more and more studies corroborated this finding (Yehuda et al., 1990, 1993; Brand et al., 2006; Rohleder and Karl, 2006; Wessa et al., 2006). However, there are studies also showing no differences (Baker et al., 1999; Duval et al., 2004; Yehuda et al., 2004a,b; Otte et al., 2007) as well as increased (Bremner et al., 2003; Inslicht et al., 2006; Lindauer et al., 2006) levels of basal cortisol in PTSD patients. Because this topic remains controversial in the literature, more insights such as those presented in Zoladz and Diamond (2013) are recommended. Alterations in other compartments of the HPA axis were also identified in PTSD patients such as enhanced GR sensitivity and HPA axis enhanced negative feedback (Grossman et al., 2003; Duval et al., 2004; Yehuda et al., 2004b; Rohleder and Karl, 2006), elevated levels of CRH in the cerebrospinal fluid (Bremner et al., 1997; Baker et al., 1999) and reduced release of ACTH after CRH (Smith et al., 1989) and CCK4 (Kellner et al., 2000) administration. Animal models of PTSD can directly address these questions, but it is known that different types of aversive exposure in PTSD models (e.g., predator, shock, odor) or even the controllability of stress interact in a complex neuro-gene-environment system (Daskalakis et al., 2013; Zoladz and Diamond, 2013; Homberg et al., 2016; Maier and Seligman, 2016).

Actually, exposure to severe stressors such as urban violence, sexual abuse, combat in war, disasters and many others, is believed to be associated to development of PTSD, leading individuals to present physiological and behavioral alterations including nightmares, hypervigilance, flashbacks of the trauma and sleep disturbances (DSM-V, Zoladz and Diamond, 2013; Yehuda et al., 2015). Although a positive correlation has been reported between the severity of the trauma and PTSD symptomatology in veteran soldiers (Snow et al., 1988; Sutker et al., 1993), as well as in civilians (Shore et al., 1986; Pynoos et al., 1993; Mollica et al., 1998), this is not always the case (Başoglu et al., 1994; Schnyder et al., 2001; Stevenson et al., 2001). It has also been reported that the type of stressor seems to play a bigger role than its severity (Kessler et al., 1995; Amir et al., 1996).

Interestingly, not everybody develops PTSD after trauma exposure, which suggests that other factors (despite the trauma severity and type), such as socioeconomic profile, psychiatry disorder history, substance abuse, immune system, genetics and epigenetics play a role on the susceptibility for PTSD. Actually, there are evidences showing that the interplay between environment and genetics (and epigenetics) are risk factors for PTSD development (Mehta and Binder, 2012; Wilker and Kolassa, 2012; DiGangi et al., 2013).

In patients with epilepsy, stress is usually reported as one of the major seizure precipitants (Frucht et al., 2000; Spector et al., 2000; Nakken et al., 2005; Sperling et al., 2008; van Campen et al., 2012; Ferlisi and Shorvon, 2014). In adults, there is a positive correlation between stress and frequency of epileptic seizures (Swinkels et al., 1998; Moshe et al., 2008). Similarly, children with epilepsy living close to conflict/war areas have a higher frequency of seizures in comparison to children living in areas without conflict (Bosnjak et al., 2002). Epileptogenesis is a multi-stage process that can begin early in life and may be negatively influenced by stress (Joëls, 2009), and it is now suggested that early life stress can create permanent vulnerability to the development of epilepsy (Huang, 2014).

Therefore, many evidences indicate that stress is relevant in the phases of epileptogenesis, both in adults and young people. Corticosterone plays a contributory role in the epileptogenic process in animal models of epilepsy (Karst et al., 1999; Kumar et al., 2007; Hemanth Kumar et al., 2012; Salzberg et al., 2007; Koe et al., 2009; Castro et al., 2012; Jones, 2013; van Campen et al., 2014), and there are several actions of corticosterone in the brain that could mediate such effects. This could occur indirectly through the genomic effects of glucocorticoids, resulting in alterations of networks associated with seizures, or by direct non-genomic effects of these on the excitability of the limbic system (Joëls, 2009).

Other pathologies affect the peripheral nervous system and many different organs. The CRH circuitry connecting LC-NE and PVN is strategically positioned so it may control autonomic responses to visceral stimuli and may underlie the co-morbidity of pelvic visceral and behavioral symptoms observed in many stress-related disorders (Valentino et al., 1999). Although most immediate responses coordinated by SAM axis are important for survival, when levels of circulating catecholamines are maintained elevated for prolonged periods of time, they can lead to different pathologies. Those pathologies may primarily affect the cardiovascular system (cardiac arrhythmias, angina, congestive, heart failure, hypertension and/or cardiac hypertrophy; Antunes-Rodrigues et al., 2005; Lundberg, 2005; McEwen, 2007; Dünser and Hasibeder, 2009; Zhang and Anderson, 2014; Carter and Goldstein, 2015; Tank and Lee Wong, 2015; Breen et al., 2016).

In the past decades identification of neuronal circuits associated to stress, as well as their interaction with mediators over time, was critical not only for understanding physiological stress responses, but also to understand their clinical implications. Stress-related brain disorders are extremely prevalent, so identification of mechanisms related to stress and consequently the potential development of new pharmacological therapeutic approaches are necessary and urgent.

As an example, pre-clinical studies on psychopathology-related topics are being done with classic GR antagonist RU486 (Arp et al., 2016) as well as using newly developed compounds, which modulate the GR in a tissue-gene specific fashion. These compounds are the so-called selective glucocorticoid receptor modulators (SGRMs) which upon binding to the GR promote a differential conformation of the receptor leading to differential recruitment of corregulators and therefore enhancing or hampering gene expression in a cell-tissue specific manner. Thus, the same compound can induce agonist- and antagonist-like effects, for instance the SGRMs C108297 acts as an antagonist in neurogenesis related processes such as proliferation and survival of hippocampal neurons, and as an agonist in fear memory retention on the avoidance behavior task (Zalachoras et al., 2013). In the other hand the C118335 compound acts as an antagonist in the same task whereas showing agonist effects on plasmatic corticosterone levels (Atucha et al., 2015).

From our own experience the Wistar Audiogenic Rat (WAR) strain is a genetically selected experimental epilepsy model, which displays after 56 generations of inbreeding, not only seizure-associated behaviors and electrophysiological alterations, but also comorbidities which includes high anxiety, hyperactive HPA axis, adrenal medulla hyperplasia, ectopic beats, high blood pressure, tachychardia and central respiratory alterations (Fazan et al., 2011, 2015; Umeoka et al., 2011; Granjeiro et al., 2016; Totola et al., 2017), for a recent comprehensive review on the WAR strain see Garcia-Cairasco et al. (2017). Further experiments are therefore needed in order to evaluate the pharmacological profile of new anti-epileptic, anxiolytic or even anti-depressive drugs, using as a model of comorbidities of the WAR strain.

In the clinical scenario a final comment can be done, when referring to the additional complexity associated, for example, to the presence of comorbid neurological and neuropsychiatric conditions, such as, for example, the epilepsies, autism and mood disorders, recognizing that we are talking about network disorders (Kanner et al., 2017). In the particular case of depression, anxiety and epilepsy, Kanner (2017) and Nogueira et al. (2017) highlight how strong are the bidirectional links that exist between epilepsy and depression, anxiety and epilepsy. Therefore stressors, which work as triggers, such as those cited in the current review, are common to all the situations. On the other hand, Rayner (2017) highlights that complex cognitive networks associated to depression interact so strongly with epilepsy related networks, in a way that the difficulties with diagnosis and treatment increase, as soon as we recognize that those networks share common structures and mechanisms. One way to overcome this, in order to make relevant and reliable contributions in this field, is the construction of algorhythms from computational neuroscience modeling, where actual data, either from basic science or from human clinical settings are used to generate predictions with translational value. In that context, recently Spiga et al. (2017) developed a mathematical model of the adrenal steroidogenic regulatory network that accounts for key regulatory processes occurring at different timescales and using the model to predict the time evolution of steroidogenesis in response to physiological ACTH alterations (basal pulses vs. inflammatory stress). In brief, these authors showed in a rat model, that although the steroidogenic regulatory network architecture is sufficient to respond to both small and large ACTH perturbations, coupling the regulatory network with the immune system would explain dissociated dynamics between ACTH and glucocorticoids observed when inflammatory stress is present.

## Concluding Remarks

We are able to adapt to the dynamic and challenging environment we live in, as well as to unexpected life events we face every now and then. Examples are endless and, most of the time, we can overcome these events. We are able to do so because the existence of quite complex networks, which integrate body and brain, in order to enhance performance, promote adaptation and ultimately survival, *the stress system*.

A diversity of brain areas integrates sensorial, physiological and emotional signs. When different brain networks interpret these signs as a threat (real or potential), a series of responses follow, increasing performance to deal with the situation and retain that information to better cope with similar situations in the future, characterizing *the stress response*. The stress response, by means of mediator molecules, promotes short and long-term alterations in cellular excitability, as well as in neuronal and synaptic plasticity leading to transient and/or permanent changes in physiology and behavior.

The downside of the stress system is that sometimes it is not able to overcome the environmental, physiological or emotional demand. It mostly happens in occasions in which the demand is extremely strong, chronic and/or during development.

The aim of this review article was to bring comprehensive basic concepts about the stress system such as history of stress research, neuroanatomy, major effectors of the stress response, time domains of stress and the clinical implication of malfunction might have over the susceptibility to the development of increasingly-common brapdin disorders.

## Author Contributions

LDG, MTR and PD-P wrote the manuscript draft. NG-C and EHLU conceptualize, wrote and corrected the manuscript. LDG and MTR contributed equally to this work.

## Conflict of Interest Statement

The authors declare that the research was conducted in the absence of any commercial or financial relationships that could be construed as a potential conflict of interest.
